# Real-life cohort experience after implementing HIV pre-exposure prophylaxis for one year in northwest Spain

**DOI:** 10.3389/fpubh.2022.1005622

**Published:** 2022-10-28

**Authors:** Alexandre Pérez-González, Marta Represa, Pep Coll, Carmen Potel, Silvia Rodríguez-Rivero, Erene V. Flores, Claudia Vázquez-Estévez, Antonio Ocampo, Guillermo Pousada, Eva Poveda

**Affiliations:** ^1^Group of Virology and Pathogenesis, Galicia Sur Health Research Institute (IIS Galicia Sur), Complexo Hospitalario Universitario de Vigo, SERGAS-UVigo, Vigo, Spain; ^2^Infectious Diseases Unit, Internal Medicine Department, Complexo Hospitalario Universitario de Vigo, Vigo, Spain; ^3^IrsiCaixa AIDS Research Institute, Barcelona, Spain; ^4^Infectious Diseases Department, Hospital Universitari Germans Trias i Pujol, Barcelona, Spain; ^5^Microbiology Department, Complexo Hospitalario Universitario de Vigo, Vigo, Spain; ^6^Anal Dysplasia Unit, General Surgery Department, Complexo Hospitalario Universitario de Vigo, Vigo, Spain; ^7^Infectious Diseases Group, Galicia Sur Health Research Institute (IIS Galicia Sur), Vigo, Spain

**Keywords:** HIV, pre-exposure prophylaxis (PrEP), sexually transmitted infections, *Chlamydia trachomatis*, *Neisseria gonorrhoeae*, human papillomavirus (HPV)

## Abstract

**Introduction:**

Pre-exposure prophylaxis (PrEP) has become a useful tool to reduce the transmission of human immunodeficiency virus (HIV) in key populations. In this article we assessed the effectiveness, safety, adherence, sexually transmitted infections (STIs) dynamics, and frequency of anal dysplasia among a real-life cohort of PrEP users in Northwest Spain.

**Methods:**

A retrospective cohort study was undertaken in the Alvaro-Cunqueiro Hospital, Vigo which included every individual who started daily emtricitabine/tenofovir-disoproxil-fumarate (FTC/TDF) between November-2019 and October-2021. Clinical and epidemiological data were obtained from the patient's medical records. The effectiveness and safety of FTC/TDF were assessed by HIV serology and renal function monitoring every 3 months. Anal, urethral, and oropharyngeal exudates were collected quarterly after the baseline visit.

**Results:**

A total of 126 individuals were considered eligible, most of the participants had previously been diagnosed with a STI (60.3%), 22% had consumed recreational drugs in the year prior, and 13% had engaged in chemsex. At the end of the follow-up, no cases of HIV infection were detected; 3 patients had discontinued FTC/TDF because of side effects but none of them had presented renal toxicity. In addition, the diagnosis of STIs during the follow-up was common (100 cases in 54 patients). Moreover, engagement in chemsex was more common within this latter group (22 vs. 6%, *p* = 0.013). Among the study population included in the anal screening programme, the frequency of dysplasia was 9%.

**Conclusions:**

FTC/TDF was effective, safe, and tolerable in a real-life cohort; adherence remained high throughout the study period (79%). However, a high number of STIs were diagnosed, especially among patients who engaged in chemsex.

## Introduction

In recent years pre-exposure prophylaxis (PrEP) has become a useful strategy to reduce the transmission of human immunodeficiency virus (HIV) (1). PrEP regimens are based on daily or on-demand administration of antiretroviral drugs (e.g., emtricitabine or tenofovir) in key populations such as men who have sex with men (MSM) or sex workers. The combination of emtricitabine (FTC) and tenofovir-disoproxil-fumarate (TDF) has been proven to reduce the transmission of HIV (2), both when used as daily or on-demand regimens (3). In addition, FTC/TDF showed a good safety profile in previous studies. Nonetheless, some concerns have been raised in relation to a possible increase in the expression of kidney tubule health biomarkers associated with the use of these drugs (4).

Whether FTC/TDF is related to a decline in the estimated glomerular filtration rate (eGFR) remains controversial and so far, contradictory data has been reported in this respect (4, 5). Importantly, kidney tubule and bone toxicity are a common side effect of TDF-based regimens among people living with HIV (PLWH) (6). However, a novel formulation of TDF, tenofovir-alafenamide (TAF), seems to present a safer renal profile (7) and may be more appropriate for future use. Moreover, in a clinical trial involving PrEP users, patients receiving an FTC/TAF regimen showed better renal biomarker profiles compared to those using FTC/TDF (8).

Nevertheless, in the context of PrEP, some concerns have emerged regarding the incidence of sexually transmitted infections (STIs) other than HIV. In this respect, several studies have reported a high incidence of STIs among PrEP users (9), a decrease in condom usage (10), and an increase in the consumption of recreational drugs during sexual encounters (chemsex) (11). Furthermore, human papillomavirus (HPV) infection is more common among MSM, especially those infected by HIV (12), for whom specific anal dysplasia screening programs have been developed in recent years (13). Whether these programs are useful for HIV-uninfected MSM remains unknown (14). Therefore, real-life data is critical to better understand the safety and effectiveness of FTC/TDF, STI dynamics, and the prevalence of anal dysplasia among PrEP users and other key populations (e.g., MSM and sex workers, among others).

In November 2019, an FTC/TDF based PrEP programme was implemented by the Public Health Service in Northwest Spain (Galicia). This plan included the prescription of a daily dose of FTC/TDF, kidney function monitoring, and STI screening every 3 months for individuals at a high risk of HIV infection. Herein, we report our experience with a real-life cohort located in Northwest Spain, including data from the time the use of PrEP was initiated in our health area. The PrEP effectiveness, safety, sexual behaviours, drug consumption, and STI incidence during the study period were all reviewed.

## Methods

### Study design

An observational retrospective cohort study was undertaken by the Infectious Diseases Department in the University Hospital Complex of Vigo which has a catchment area population of 450,000 inhabitants. The medical records of individuals who requested PrEP administration were reviewed. The Galician PrEP programme (15) started in November 2019 and included MSM and transgender women for whom at least two of the following criteria applied: regular condomless sex, more than 10 sexual partners in the year prior, recreational drug consumption during sexual intercourse, HIV post-exposure prophylaxis prescribed at least twice in the year prior, or a STI diagnosed in the year prior. All the patients completed a baseline visit which included STI screening and HIV serology prior to the FTC/TDF administration. Once HIV-infection was ruled out, daily FTC/TDF was prescribed and from thereon the kidney parameters, HIV status, and presence of STIs were monitored in these patients every 3 months.

### Sample collection and laboratory tests

Samples were processed at the Microbiology Service at the University Hospital Complex of Vigo according to laboratory standards. Anal, urethral, and oropharyngeal samples were screened for *Chlamydia trachomatis* (CT) and *Neisseria gonorrhoeae* (NG) using a Cobas^®^ 6,800 system (Roche, Switzerland). Serum samples were tested for HIV (Cobas HIV-1/HIV-2 Qual; Roche), *Treponema pallidum* (Chemiluminescence Immunoassay, Liason Treponema Screen, LIAISON, Diasorin, Italy Rapid Plasma Reagin, BioMérieux^®^, France), and hepatitis C virus (HCV; Cobas 6,800/8,800 HCV, Roche). The Roche Cobas HPV test was used to detect high-risk human papillomavirus (HPV) in anal cytology samples and to genotype HPV-16 and HPV-18 as well as a grouped category of high oncogenic risk HPV (pHR-HPV; 31, 33, 35, 39, 45, 51, 52, 56, 58, 59, 66, and 68). The anal cytology was processed in the Pathology Department according to routine procedures and later categorised based on the standard criteria. Biopsies obtained during the anoscopy were reviewed by an expert pathologist and classified according to the Bethesda criteria.

### Anal dysplasia surveillance

At the time of their baseline visit all the patients were invited to participate in the Anal Dysplasia Surveillance Programme. For those who accepted the offer, an anal cytology sample was obtained during the anal exam. A high-resolution anoscopy (HRA) was then conducted by an expert surgeon to screen for altered anal exam results or abnormal cytology results (e.g., the identification of condyloma acuminata, i.e., genital warts).

### Variable definitions

A past STI was defined as any STI diagnosed prior to the inclusion in the PrEP programme; baseline STI status was defined as a venereal disease diagnosed during the baseline visit (prior to FTC/TDF prescription); and finally, an incident STI was defined as a new episode of a STI that occurred during the follow-up after starting PrEP.

### Statistical analyses

The study data were collected and managed using REDCap electronic data capture tools hosted at the *Instituto de Investigación Sanitaria Galicia Sur*. Quantitative variables were expressed as the median and interquartile range. Qualitative variables were shown as absolute values and percentages. Categorical variables were compared using χ-squared tests or the Fischer exact test, as appropriate. Quantitative variables were compared by employing Man–Whitney *U*-tests. *P*-values < 0.05 were considered significant in all cases. The statistical analyses were performed using Statistical Package for Social Sciences (SPSS) software (version 22, IBM Corp., Armonk, NY).

### Ethics

This study was approved by the Ethics Committee of Pontevedra-Vigo-Ourense (reference 2021/311). The need for informed consent was waived because of the retrospective design of this work. The STROBE guidelines were used to ensure the reporting of this study.

## Results

During the study period, a total of 136 individuals requested the prescription of PrEP in the Infectious Disease Unit. Of these, 10 were excluded in the final study cohort, in 6 cases because the patients had been included in a clinical trial, 3 individuals tested positive for HIV-infection, and 1 patient eventually declined to use PrEP. Thus, a total of 126 patients were finally considered eligible for this study. The baseline characteristics of the study population are shown in Table 1. Briefly, most of the participants were young males (median age 35 years), 22.2% (*n* = 28) had consumed recreational drugs in the year prior, and 12.7% (*n* = 16) had consumed recreational drugs during sex (chemsex). Most of patients had previously been diagnosed with an STI, with syphilis being the most frequent (32.5%, *n* = 41).

**Table 1:** Baseline characteristics of the study population.

**Total study population**	***N* = 126**
Male, *n* (%)	124 (98.4%)
Transgender woman, *n* (%)	2 (1.6%)
Age, mean, in years	35.0 (9.0)
**Ethnicity**
Spanish, *n* (%)	102 (80.9%)
Latin American, *n* (%)	22 (17.5%)
Other, *n* (%)	2 (1.6%)
**Tobacco consumption**
Active smoker, *n* (%)	36 (28.6%)
Former smoker, *n* (%)	5 (3.9%)
Never smoker, *n* (%)	54 (42.9%)
Unknown, *n* (%)	31 (24.6%)
Drug consumption last year	28 (22.2%)
Chemsex	16 (12.7%)
Intravenous drugs (*slamming*)	1 (0.8%)
Cocaine	12 (9.5%)
Cannabis	8 (6.3%)
Mephedrone	8 (6.3%)
Alkyl nitrites (*poppers*)	5 (4.0%)
PDE5-inhibiting drugs (e.g., sildenafil)	3 (2.4%)
Gamma-Hydroxybutyric acid and derivates (e.g., *GHB or GBL*)	3 (2.4%)
Past STI diagnosis, *n* (%)	76 (60.3%)
Syphilis, *n* (%)	41 (32.5%)
*Neisseria gonorrhoeae* infection, *n* (%)	27 (21.4%)
Condyloma acuminata (genital warts), *n* (%)	20 (15.9%)
*Chlamydia trachomatis* infection, *n* (%)	15 (11.9%)
Other (genital herpes, scabies, etc.), *n* (%)	12 (9.5%)
Active HCV infection at baseline	0
**Sexual role**
Versatile	61 (48.4%)
Passive	10 (7.9%)
Active	9 (7.1%)
Unknown	46 (36.5%)
Age at the time of first sexual intercourse, in years	17 (3)
Number of sexual partners (in prior year)	15 (12)
Number of sexual partners (lifetime)	100 (250)
Condom use during sexual intercourse	9 (7.1%)
Received PEP at least once	11 (8.7%)

Quantitative variables are expressed as the median and interquartile range; qualitative variables are expressed in numbers and percentages. PDE5, phosphodiesterase type 5; STI, sexually transmitted infection; HCV, hepatitis C infection; PEP, HIV post-exposure prophylaxis.

### Baseline screening

According to the local protocols, a baseline STI screening was performed prior to the administration of FTC/TDF. Three patients tested positive for HIV-infection and were therefore excluded from the PrEP programme. An acute HIV-infection was suspected in the first patient (he referred high-grade fever and odynophagia) while the other two individuals were asymptomatic; two were born in Latin America and the other was born in Spain. The ages were 19, 24, and 28 years old, two of them had a previous history of a STI (one syphilis case and one gonorrhoea, respectively). None of them had previously received post-exposure prophylaxis (PEP). All three patients had tested negative for HIV-infection in the prior year. No HCV infection cases were detected. Among the remaining patients, 30.2% (*n* = 38) tested positive for a bacterial STI, with CT (*n* = 17, 13.5%) and NG (*n* = 15, 11.9%) being the pathogens most commonly isolated.

### The safety and effectiveness of pre-exposure prophylaxis

The median follow-up time was 13 months, and no cases of HIV or HCV infections were detected. At the end of the study period 79% (*n* = 100) of the participants remained on PrEP, 14% (*n* = 17) were lost to follow-up, and PrEP was no longer indicated (i.e., the patient had a stable sexual partner or consistently used condoms) in 5% (*n* = 6) of the cases. FTC/TDF was discontinued because of safety or toxicity concerns in 3.2% (*n* = 3) individuals, 2 of them because of digestive intolerance and 1 as the result of skin toxicity (severe folliculitis attributed to the drugs). No patients stopped FTC/TDF treatment due to kidney toxicity.

### Risk factors for incident sexually transmitted infections

**Figure 1:**
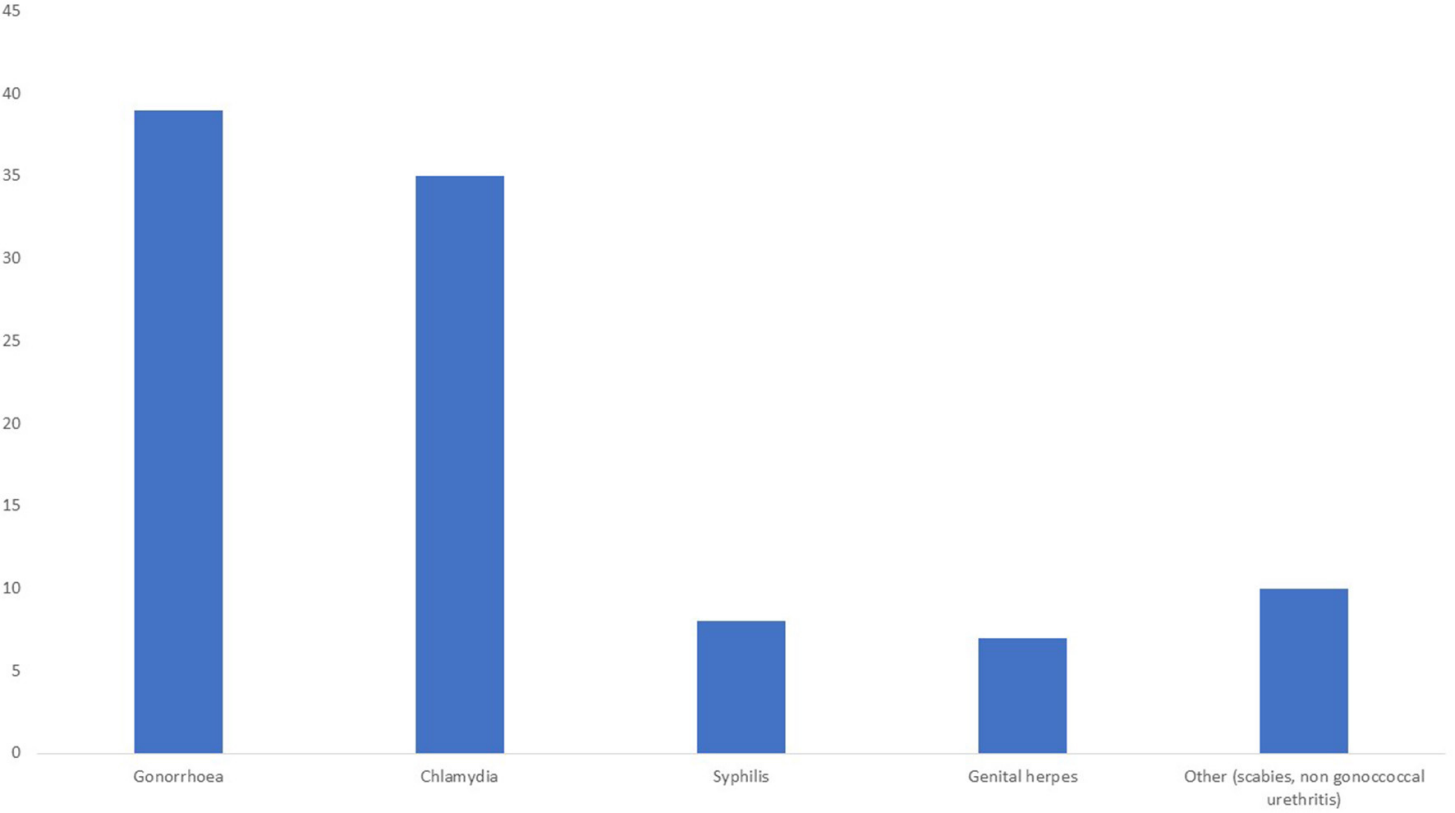
Total number of sexually transmitted diseases detected during the follow-up period.

**Figure 2:**
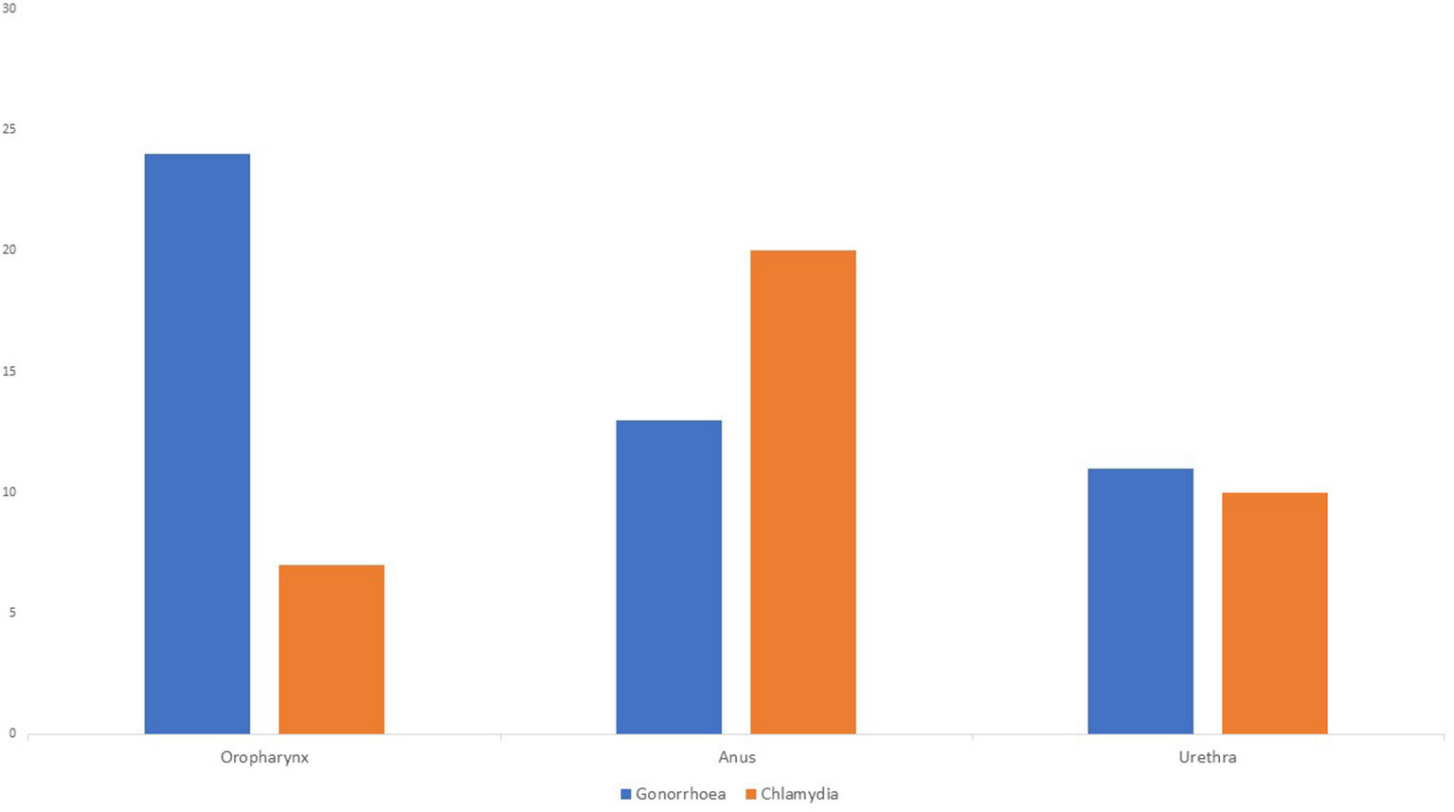
Total number of *Chlamydia trachomatis* and *Neisseria gonorrhoeae* cases according to the anatomical location.

During the follow-up, 43% of patients (*n* = 54) were diagnosed with at least one STI, with NG (39%, *n* = 39) and CT (35%, *n* = 35) infections accounting for most cases (Figure 1), although the anatomical locations affected slightly differed. NG was most often isolated from oropharyngeal samples while CT was more commonly found in the anal region (Figure 2). Syphilis was less common (8%, *n* = 8) and the majority of cases were of early asymptomatic and latent syphilis (*n* = 7); only one patient presented a chancre. In addition, genital herpes was detected in *n* = 7 individuals, while other STIs (scabies or non-gonococcal urethritis) were diagnosed in 10 patients.

Patients were classified into two groups according to whether an incident STI was detected or not; the comparison between these two groups is shown in Table 2. Recreational drug consumption was more common in the STI group (33.3 vs. 13.8%, *p* = 0.017), especially among those who practiced chemsex (22.2 vs. 5.5%, *p* = 0.013). Patients who were diagnosed with an STI at the baseline visit exhibited a higher proportion of incident STIs, although this difference was not statistically significant (38.9 vs. 23.6%, *p* = 0.064). Conversely, the age of the first sexual encounter, number of sexual partners, and prior prescription of a post-exposure prophylaxis (PPE) treatment, did not differ between the two groups.

**Table 2:** Univariant analysis of the incidence of sexually transmitted infections risk factors.

	**Incident STI *n* = 54 (42.9%)**	**Non-incident STI *n* = 72 (57.1%)**	***P*-value**	**RR (95% CI)**
Age at the time of PrEP initiation, in years	37 (9)	34 (15)	*p* = 0.900	
Age at the time of first sexual intercourse, in years	17.5 (4)	17.0 (2)	*p* = 0.773	
Number of sexual partners in the year prior	16.5 (10)	15.0 (19)	*p* = 0.340	
Recreational drug consumption	18 (33.3%)	10 (13.8%)	***p*** **=** **0.017**	1.7 (1.2–2.5)
Chemsex	12 (22.2%)	4 (5.5%)	***p*** **=** **0.013**	1.9 (1.3–2.8)
Baseline STI	21 (38.9%)	17 (23.6%)	*p* = 0.064	
Received PPE at least once	4 (7.4%)	7 (9.7%)	*p* = 0.757	

Quantitative variables are expressed as the median and interquartile range; qualitative variables are expressed in numbers and percentages. PrEP, pre-exposure prophylaxis; RR, risk ratio; STI, sexually transmitted infection; PPE, HIV post-exposure prophylaxis. Statistically significant *p* values were highlighted with bold letters.

### Human papillomavirus infection and anal dysplasia

During the baseline visit, all the patients were invited to join an anal squamous intraepithelial lesion (SIL) surveillance programme, with 77% (*n* = 97) of the participants accepting this offer (Table 3). Abnormal cytology results were detected in 23% (*n* = 22) individuals and HRA was performed in *n* = 26; 9.3% (*n* = 9) patients were diagnosed with an anal SIL, with most of them being low-grade (LSIL) except for 1 high-risk (HSIL) case. The prevalence of HPV within this subgroup was common (69.1%), in most of these cases due to pHR-HPV (55.7%), with HPV-16 (17.5%) and HPV-18 (3.1%) diagnoses being less usual. Most patients (86%) had received the nine-valent HPV vaccine prior to their inclusion in the anal dysplasia screening programme.

**Table 3:** Prevalence of human papillomavirus infection in the patients included in the anal squamous intraepithelial lesion surveillance programme.

**Anal SIL sub study**	***n* = 97**
HR-HPV	67 (69.1%)
HPV-16	17 (17.5%)
HPV-18	3 (3.1%)
pHR-HPV	54 (55.7%)
Abnormal anal cytology result	22 (22.7%)
HRA performed	26 (26.8%)
Anal dysplasia	9 (9.3%)
LSIL	8 (8.2%)
HSIL	1 (1.0%)

SIL, squamous intraepithelial lesion; HPV, human papillomavirus; HR-HPV, high oncogenic risk HPV; pHR-HPV, high oncogenic risk (grouped); HRA, high-resolution anoscopy; LSIL, low-grade squamous intraepithelial lesion; HSIL, high-grade squamous intraepithelial lesion.

## Discussion

PrEP has become a useful public health tool to reduce the transmission of HIV in key populations. Thus, several health systems, including those in Spain, have developed PrEP programmes in recent years. In addition to the primary objective of HIV prevention, PrEP programs might serve as useful tools to monitor other public health concerns (e.g., STIs or the prevalence of chemsex). Real-life studies also provide valuable data concerning the safety and effectiveness of FTC/TDF and STI dynamics after the implementation of PrEP programmes worldwide. To the best of our knowledge, this is the first study to report real-life data after the implementation of a PrEP programme in Northwest Spain. In this cohort, FTC/TDF was highly effective with no HIV infections detected during the study period and no treatment discontinuations because of renal toxicity. The main cause for PrEP discontinuation in our study population was the presentation of adverse gastrointestinal events (2.4%); interestingly, these were also more common in the FTC/TDF arm compared to the placebo (14 vs. 5%, respectively) in the IPERGAY clinical trial (2). Adverse skin side-effects were also less frequent than digestive disruptions (< 1%), accounting for around 1.7 cases per 1,000 person-years in the United States within PrEP users (16).

In the recent years, access to HIV, STI and PrEP care has become more challenging owed to the COVID-19 pandemic (17, 18). Nevertheless, among our study population, only 31 individuals were on PrEP by 15 March 2020, when a national lockdown was imposed in Spain owed to the COVID-19 pandemic. Those patients included in the programme before the pandemic, remained attending to the unit as usual.

A major concern within key populations is an increasing incidence of different STIs (15). It is concerning that, despite the COVID-19 pandemic, the frequency of STIs continues to increase (19) and the widespread presence of multi-drug resistant sexually transmitted pathogens has been reported (20). Moreover, there is a higher incidence of STIs among PrEP users (21) because condom use is less frequent among this group. Of note, *Chlamydia trachomatis* infection was frequently isolated from anal samples, which has been described as a main risk factor for HIV acquisition (22), thus those patients diagnosed of anal *Chlamydia* might deserve special attention owed to an increased risk of HIV infection Anal HPV infection is also common among MSM, although data concerning its prevalence among PrEP users is scarce. However, a sub study in the IPERGAY clinical trial revealed a higher prevalence of anal HPV infection compared to our study population (92 vs. 69%, respectively), including infection with HPV-16 (22 vs. 18%) (23). Of note, most of our patients (86%) had received an HPV vaccine prior to or during their baseline visit. The main concern regarding HPV infection is the oncogenic potential of this virus to induce several anogenital cancers (e.g., cervical, anal, etc.) (24).

In addition, SIL surveillance programs have been developed for cervix and anal cancer prevention which have led to a decline in the incidence of HPV-driven dysplasia. Anal squamous cell carcinoma is a rare disease among the general population, but its incidence is disproportionally high in HIV-infected MSM (25). Whether anal SIL surveillance is necessary among HIV-uninfected MSM remains controversial, and very little data are available in this respect (14). Owed to our previous experience in PLWH (13), PrEP users were invited to a join an HPV surveillance program which revealed a SIL prevalence of 9% in our cohort, mostly comprising LSIL cases. Nonetheless, prospective studies will be required to assess the usefulness of anal screening among PrEP users. Meanwhile, HPV vaccination should be encouraged as the main preventive tool for anal dysplasia.

Of note, chemsex has emerged in recent years as a major concern both among PLWH and PrEP users. Chemsex is associated with the consumption of various psychoactive recreational drugs (e.g., gamma-hydroxybutyric acid, mephedrone, or ecstasy), group sex, and condomless intercourse that can last hours or even days (26). The overall prevalence of chemsex participation among MSM varies widely with one meta-analysis estimating an overall prevalence of 2–28% (27). Conversely, Ruiz-Robledillo et al. reported higher rates of chemsex among PrEP users in Spain at 40.6 and 63%, respectively (28, 29). However, chemsex was less frequent (13%) in our study population and only 1 patient reported intravenous recreational drug usage.

Chemsex is also associated with harmful effects in terms of mental health, addiction, and an increased risk of STI/HIV acquisition. Depression, anxiety, and poorer mental health scores are all more common among people who practice chemsex (30). Moreover, the incidence of STIs is also higher among chemsex users (31) with some studies suggesting an increased risk of HIV infection because of a high rate of condomless sexual intercourse among this group (32). Regarding our study population, we detected an increased risk of incident STIs among chemsex users (22.2 vs. 5.5%), although no HIV or HCV infections occurred. Conversely, chemsex was not related to poorer compliance with PrEP regimens (33). Indeed, chemsex users within our study population exhibited similar compliance rates to those who did not engage in chemsex (87.8 vs. 84.6%).

Finally, it is important to note that our study had several limitations. Firstly, the retrospective design of the work might have resulted in some data loss. Our sample size was limited, likely because of the COVID-19 lockdown and social restrictions that may have reduced the demand for PrEP regimens. In addition, in our health area, the implementation of PrEP is restricted to daily treatments while on-demand use of FTC/TDF is discouraged, which might dissuade some patients from using it. Secondly, the sexual habits of the participants (i.e., number of sexual partners and condom usage, etc.) were only recorded at the baseline visit, not during the use of PrEP. Thus, any behavioural changes during the follow-up period were not considered in this current work. Thirdly, no renal biomarkers for TDF kidney toxicity (i.e., proteinuria, phosphaturia, etc.) were measured, although no patients stopped using TDF for renal safety reasons. Finally, at our centre, the screening samples were not routinely tested for *Mycoplasma genitalium* (an emerging sexually transmitted pathogen), and CT isolates were not tested for lymphogranuloma venereum *Chlamydia trachomatis* L1–L3 serovars.

## Conclusions

In our study population, the daily use of FTC/TDF was effective and safe. Most patients continued the follow-up for 1 year and treatment discontinuations due to adverse effects were uncommon. Both the incidence and prevalence of STIs were high, especially among chemsex users. Finally, the presence of HPV-driven dysplasia should not be ignored, and long-term studies will be needed to analyse its relevance among PrEP users.

## Data availability statement

The raw data supporting the conclusions of this article will be made available by the authors, without undue reservation.

## Ethics statement

This study was approved by the Ethics Committee of Pontevedra-Vigo-Ourense (reference 2021/311). Written informed consent for participation was waived for this study in accordance with the national legislation and the institutional requirements.

## Author contributions

AP-G: designed data collection tools, monitored data collection, analysed the data, and drafted the manuscript. MR, SR-R, CV-E, GP, and EF: collected data. PC: revised the manuscript. CP and AO: analysed the data and revised the manuscript. EP: designed, analysed, and revised the manuscript. All authors contributed to the article and approved the submitted version.

## Funding

AP-G principal investigator is hired under a Río Hortega contract financed by the *Instituto de Investigación Carlos III* (ISCIII) with reference number CM20/00243.

## Conflict of interest

The authors declare that the research was conducted in the absence of any commercial or financial relationships that could be construed as a potential conflict of interest.

## Publisher's note

All claims expressed in this article are solely those of the authors and do not necessarily represent those of their affiliated organizations, or those of the publisher, the editors and the reviewers. Any product that may be evaluated in this article, or claim that may be made by its manufacturer, is not guaranteed or endorsed by the publisher.

## References

[1] Sepodes B Rocha J Batista J Figueira M-E Dráfi F Torre C. Implementation and Access to Pre-exposure Prophylaxis for Human Immunodeficiency Virus by Men Who Have Sex With Men in Europe. Front Med. (2021) 8:722247. 10.3389/fmed.2021.722247 34513883 PMC8424070

[2] Molina J-M Capitant C Spire B Pialoux G Cotte L Charreau I. le Gall J-M, Cua E, Pasquet A, et al. On-Demand Preexposure Prophylaxis in Men at High Risk for HIV-1 Infection N Engl J Med. (2015) 373:2237–46. 10.1056/NEJMoa1506273 26624850

[3] Kwan TH Lui GCY Lam TTN Lee KCK Wong NS Chan DPC. Comparison between daily and on-demand PrEP (pre-exposure prophylaxis) regimen in covering condomless anal intercourse for men who have sex with men in Hong Kong: A randomized, controlled, open-label, crossover trial. J Int AIDS Soc. (2021) 24:1–9. 10.1002/jia2.25795 34473402 PMC8412015

[4] Ascher SB Scherzer R Estrella MM Shigenaga J Spaulding KA Glidden D. HIV preexposure prophylaxis with tenofovir disoproxil fumarate/emtricitabine and changes in kidney function and tubular health. AIDS. (2020) 34:699–706. 10.1097/QAD.0000000000002456 31794523 PMC7071971

[5] Liegeon G Antoni G Pialoux G Capitant C Cotte L Charreau I. Changes in kidney function among men having sex with men starting on demand tenofovir disoproxil fumarate-emtricitabine for HIV pre-exposure prophylaxis. J Int AIDS Soc. (2020) 23, e25420. 10.1002/jia2.25420/full 32086878 PMC7035456

[6] Sutton SS Magagnoli J Hardin JW Hsu L-I Beaubrun A Majethia S. Association of tenofovir disoproxil fumarate exposure with chronic kidney disease and osteoporotic fracture in US veterans with HIV. Curr Med Res Opin. (2020) 36:1635–42. 10.1080/03007995.2020.1816538 32856940

[7] Teira R Diaz-Cuervo H Aragão F Muñoz J Galindo P Merino M. eGFR-EPI changes among HIV patients who switch from F/TDF to F/TAF while maintaining the same third agent in the Spanish VACH cohort HIV. Res Clin Pract. (2021) 22:78–85. 10.1080/25787489.2021.1955197 34410219

[8] Mayer KH Molina J-M Thompson MA Anderson PL Mounzer KC de Wet JJ. Emtricitabine and tenofovir alafenamide vs emtricitabine and tenofovir disoproxil fumarate for HIV pre-exposure prophylaxis (DISCOVER): primary results from a randomised, double-blind, multicentre, active-controlled, phase 3, non-inferiority trial. Lancet. (2020) 396:239–54. 10.1016/S0140-6736(20)31065-5 32711800 PMC9665936

[9] Chemtob D Weil C Hannink Attal J Hawila E Noff Sadeh E. HIV Pre-exposure prophylaxis (PrEP) purchase patterns and STI occurrence among Israeli men: a cohort analysis. PLoS ONE. (2021) 16:e0259168. 10.1371/journal.pone.0259168 34793473 PMC8601516

[10] Ayerdi Aguirrebengoa O Vera García M Arias Ramírez D Gil García N Puerta López T Clavo Escribano P. Low use of condom and high STI incidence among men who have sex with men in PrEP programs. PLoS ONE. (2021) 16:e0245925. 10.1371/journal.pone.0245925 33539363 PMC7861516

[11] Ringshall M Cooper R Rawdah W Perera S Bannister A Nichols K. Chemsex, sexual behaviour and STI-PrEP use among HIV-PrEP users during the COVID-19 pandemic in Brighton, UK. Sex Transm Infect. (2022) 98:312. 10.1136/sextrans-2021-055216 34400576

[12] Wei F Gaisa MM D'Souza G Xia N Giuliano AR Hawes SE. Epidemiology of anal human papillomavirus infection and high-grade squamous intraepithelial lesions in 29 900 men according to HIV status, sexuality, and age: a collaborative pooled analysis of 64 studies. Lancet HIV. (2021) 8:e531–43. 10.1016/S2352-3018(21)00108-9 34339628 PMC8408042

[13] Iribarren Díaz M Ocampo Hermida A González-Carreró Fojón J Longueira Suárez R Rivera Gallego A Casal Núñez E. Preliminary results of a screening program for anal cancer and its precursors for HIV-infected men who have sex with men in Vigo-Spain. Rev Español Enfermed Digest. (2017) 109:242–9. 10.17235/reed.2017.4274/2016 28229612

[14] Fuchs MA Multani AG Mayer KH Keuroghlian AS. Anal cancer screening for HIV-negative men who have sex with men: making clinical decisions with limited data. LGBT Health. (2021) 8:317–21. 10.1089/lgbt.2020.0257 34030486 PMC8252895

[15] Tuddenham S Hamill MM Ghanem KG. Diagnosis and treatment of sexually transmitted infections: a review. JAMA. (2022) 327:161–72. 10.1001/jama.2021.23487 35015033

[16] Kosche C Para A Brieva J West DP Palella FJ Nardone B. Incidence of cutaneous adverse events after exposure to tenofovir–emtricitabine in HIV-uninfected vs HIV-infected patients: pharmacovigilance within a large Midwestern U.S. patient population from the research on adverse drug events and reports program. J Eur Acad Dermatol Venereol. (2019) 33:e470–1. 10.1111/jdv.15799 31301245

[17] Rogers BG Tao J Maynard M Chu C Silva E Toma E. Characterizing the impact of COVID-19 on pre-exposure prophylaxis (PrEP) care. AIDS Behav. (2021) 25:3754–7. 10.1007/s10461-021-03337-2 34114166 PMC8191705

[18] Pampati S Emrick K Siegler AJ Jones J. Changes in sexual behavior, PrEP adherence, and access to sexual health services because of the COVID-19 pandemic among a cohort of PrEP-using MSM in the South. J Acquir Immune Defic Syndr 1988. (2021) 87:639–43. 10.1097/QAI.0000000000002640 33512848 PMC8533029

[19] Pagaoa M Grey J Torrone E Kreisel K Stenger M Weinstock H. Trends in nationally notifiable sexually transmitted disease case reports during the US COVID-19 pandemic, January to December 2020. Sex Transm Dis. (2021) 48:798–804. 10.1097/OLQ.0000000000001506 34224523 PMC8459909

[20] Carballo R Povoa MC Abad R Navarro C Martin E Alvarez M. Large increase in azithromycin-resistant neisseria gonorrhoeae in Northern Spain. Microb Drug Resist. (2022) 28:81–6. 10.1089/mdr.2020.0594 34402689

[21] Traeger MW Cornelisse VJ Asselin J Price B Roth NJ Willcox J. Association of HIV preexposure prophylaxis with incidence of sexually transmitted infections among individuals at high risk of HIV infection. JAMA. (2019) 321:1380. 10.1001/jama.2019.2947 30964528 PMC6459111

[22] Barbee LA Khosropour CM Dombrowksi JC Golden MR. New human immunodeficiency virus diagnosis independently associated with rectal gonorrhea and chlamydia in men who have sex with men. Sex Transm Dis. (2017) 44:385–9. 10.1097/OLQ.0000000000000614 28608786 PMC5481158

[23] Cotte L Veyer D Charreau I Péré H Cua E Carette D. Prevalence and incidence of human papillomavirus infection in men having sex with men enrolled in a pre-exposure prophylaxis study: a sub-study of the agence nationale de recherches sur le SIDA et les hépatites virales “intervention préventive de l'exposition aux risques avec et pour les hommes gays” trial. Clin Infect Dis. (2021) 72, 41–49. 10.1093/cid/ciaa002 31907521

[24] Araldi RP Sant'Ana TA Módolo DG de Melo TC Spadacci-Morena DD de Cassia Stocco R. The human papillomavirus (HPV)-related cancer biology: an overview. Biomed Pharmacother. (2018) 106:1537–56. 10.1016/j.biopha.2018.06.149 30119229

[25] Pérez-González A Cachay E Ocampo A Poveda E. Update on the epidemiological features and clinical implications of human papillomavirus infection (HPV) and human immunodeficiency virus (HIV) coinfection. Microorganisms. (2022) 10:1047. 10.3390/microorganisms10051047 35630489 PMC9147826

[26] Chone JS Lima SVMA Fronteira I Mendes IAC Shaaban AN Martins M. Factors associated with chemsex in Portugal during the COVID-19 pandemic. Rev Lat Am Enfermagem. (2021) 29:e3474. 10.1590/1518-8345.4975.3474 34468628 PMC8432586

[27] Maxwell S Shahmanesh M Gafos M. Chemsex behaviours among men who have sex with men: a systematic review of the literature. Int J Drug Policy. (2019) 63:74–89. 10.1016/j.drugpo.2018.11.014 30513473

[28] Ruiz-Robledillo N Ferrer-Cascales R Portilla-Tamarit I Alcocer-Bruno C Clement-Carbonell V Portilla J. Chemsex practices and health-related quality of life in spanish men with hiv who have sex with men. J Clin Med. (2021) 10:1662. 10.3390/jcm10081662 33924530 PMC8068924

[29] Laguno M Ugarte A Martinez-Rebollar M Sobrino Y Font G de Lazzari E. Experiencia de un programa de profilaxis preexposición en una unidad de virus de la inmunodeficiencia humana hospitalaria. Descripción del perfil basal del usuario e identificación de oportunidades de mejora. Enferm Infecc Microbiol Clin. (2021) S0213-005X(21)00178-6. 10.1016/j.eimc.2021.04.00534045097

[30] Íncera-Fernández D Gámez-Guadix M Moreno-Guillén S. Mental health symptoms associated with sexualized drug use (Chemsex) among men who have sex with men: a systematic review. Int J Environ Res Public Health. (2021) 18:13299. 10.3390/ijerph182413299 34948907 PMC8701799

[31] Flores Anato JL Panagiotoglou D Greenwald ZR Blanchette M Trottier C Vaziri M. Chemsex and incidence of sexually transmitted infections among Canadian pre-exposure prophylaxis (PrEP) users in the l'Actuel PrEP Cohort (2013–2020). Sex Transm Infect. (2022) sextrans-2021-055215. 10.1136/sextrans-2021-055215 35039437 PMC9685712

[32] Kenyon C Wouters K Platteau T Buyze J Florence E. Increases in condomless chemsex associated with HIV acquisition in MSM but not heterosexuals attending a HIV testing center in Antwerp, Belgium. AIDS Res Ther. (2018) 15:14. 10.1186/s12981-018-0201-3 30340607 PMC6195714

[33] Roux P Fressard L Suzan-Monti M Chas J Sagaon-Teyssier L Capitant C. Is on-demand HIV pre-exposure prophylaxis a suitable tool for men who have sex with men who practice chemsex? Results from a substudy of the ANRS-IPERGAY. Trial J Acquir Immune Defic Syndr. (2018) 79:e69–75. 10.1097/QAI.0000000000001781 30212434

